# Bacterial Community Dynamics and Taxa-Time Relationships within Two Activated Sludge Bioreactors

**DOI:** 10.1371/journal.pone.0090175

**Published:** 2014-03-04

**Authors:** Reti Hai, Yulin Wang, Xiaohui Wang, Yuan Li, Zhize Du

**Affiliations:** Department of Environmental Science and Engineering, Beijing University of Chemical Technology, Beijing, China; University of Erlangen-Nuremberg, Germany

## Abstract

**Background:**

Biological activated sludge process must be functionally stable to continuously remove contaminants while relying upon the activity of complex microbial communities. However the dynamics of these communities are as yet poorly understood. A macroecology metric used to quantify community dynamic is the taxa-time relationship (TTR). Although the TTR of animal and plant species has been well documented, knowledge is still lacking in regard to TTR of microbial communities in activated sludge bioreactors.

**Aims:**

1) To characterize the temporal dynamics of bacterial taxa in activated sludge from two bioreactors of different scale and investigate factors affecting such dynamics; 2) to evaluate the TTRs of activated sludge microbial communities in two bioreactors of different scale.

**Methods:**

Temporal variation of bacterial taxa in activated sludge collected from a full- and lab-scale activated sludge bioreactor was monitored over a one-year period using pyrosequencing of 16S rRNA genes. TTR was employed to quantify the bacterial taxa shifts based on the power law equation *S = cT^w^*.

**Results:**

The power law exponent *w* for the full-scale bioreactor was 0.43 (*R^2^* = 0.970), which is lower than that of the lab-scale bioreactor (*w* = 0.55, *R^2^* = 0.971). The exponents for the dominant phyla were generally higher than that of the rare phyla. Canonical correspondence analysis (CCA) result showed that the bacterial community variance was significantly associated with water temperature, influent (biochemical oxygen demand) BOD, bioreactor scale and dissolved oxygen (DO). Variance partitioning analyses suggested that wastewater characteristics had the greatest contribution to the bacterial community variance, explaining 20.3% of the variance of bacterial communities independently, followed by operational parameters (19.9%) and bioreactor scale (3.6%).

**Conclusions:**

Results of this study suggest bacterial community dynamics were likely driven partly by wastewater and operational parameters and provide evidence that the TTR may be a fundamental ecological pattern in macro- and microbial systems.

## Introduction

Biological activated sludge process is the most widely used biological process to treat municipal and industrial wastewater. The efficient and stable operation of biological wastewater treatment plant (WWTP) relies upon the relative abundance or activity of these microbial populations within it [1]. Because variations in microbial community composition are often associated with changes in the functional capabilities of those communities, understanding microbial temporal patterns of activated sludge can be critical for understanding ecosystem processes, and then to enhance the treatment performance and stability [2], [3].

In recent years, a growing collection of studies in the microbial ecology of activated sludge suggests that microbial communities of activated sludge exhibit a wide range of discernible temporal patterns, particularly within specific microbial subpopulations such as nitrifiers [4], [5], denitrifiers [6], phosphorus-accumulating organisms [7], and methanogens [8]. Such temporal variations in microbial communities are thought to be influenced by the deterministic environmental and operational variables and/or stochastic factors [9]–[11]. By contrast, in a full-scale WWTP, Kim *et al.* [12] found that some microbial communities were composed of core members that exhibit minimal temporal variability and rarer taxa that exhibit more pronounced fluctuations in abundance over time. Further, some microbial communities have the capacity to recover quickly after disturbance events, either to the pre-disturbance state or to an alternative stable state [13], [14]. Overall, these and other time series highlight that microbial communities are dynamic and exhibit temporal patterns that can reflect underlying biotic and abiotic processes.

Many previous studies have been performed in lab-scale bioreactors which treated synthetic wastewater, where selective pressures likely differ dramatically from those in full-scale plants [10]. An equilibrium model based on island biogeography also predicts that the scale of bioreactors affect microbial communities within them [3]. Indeed, a very limited number of studies have characterized microbial population dynamics in activated sludge from full-scale WWTPs. To data, there is no quantitative comparison of the change rate of microbial population within activated sludge from different scale bioreactors. Furthermore, nearly all of the previous studies employed clone library or fingerprinting methods to characterize the temporal dynamics of microbial communities in bioreactors. These techniques are lack of ability to detect rare species within a given habitat. For example, denaturing gradient gel electrophoresis (DGGE) exhibits method-dependent detection threshold in absolute abundance and it has been estimated that populations for <10^3^ individuals per sample within a given community will be below the detection threshold [11], [15]. Fortunately, the ongoing development of high-throughput sequencing technologies has made it feasible to describe the temporal dynamics of microbial communities in higher resolutions that were previously unattainable [16].

Activated sludge bioreactors are excellent test beds for fundamental questions in microbial ecology [5]. One of the fundamental objectives of ecology is to understand how biodiversity is generated and maintained across spatial and temporal scales [11], [16]. Patterns of species diversity provide important insights into the underlying mechanisms that regulate biodiversity, and are central to the development of ecological models and theories. One such pattern is the species-area relationship (SAR), which describes the tendency that species richness increases with area. SAR plays a central role in biodiversity research, and recent work has increased awareness of its temporal analogue, the species-time relationship (herein referred to as the taxa-time relationship [TTR]) [17]. The TTR describes how the species richness of a community increases with the time span over which the community is observed [18]. It is similar in form to the SAR: *S* = *cT^w^*, where *S* is the taxa richness, *T* is the time of observation, *c* is an empirically derived constant, and *w* is a scaling exponent that reflects species turnover [10]. Although the TTR of animal and plant species has been well documented [18], it is only recently that TTRs have been tested in a limited number of microbial systems [10], [16], [19]. To data, very few studies have applied the power-law TTR to activated sludge bioreactors, and they found that the accumulation of observed taxa within the bioreactor followed a power law relationship [10], [11]. However, Comparisons of the temporal scaling exponent (*w*) among various microbial populations and between bioreactors at different scale have not yet been explored.

The primary objectives of this study were thus two-fold: 1) to characterize the temporal dynamics of bacterial taxa in activated sludge from two bioreactors of different scale and investigate factors affecting such dynamics; and 2) to evaluate the TTRs of activated sludge microbial communities in two bioreactors of different scale, and compare the temporal scaling exponent *w* among different microbial populations within the two bioreactors.

## Materials and Methods

### Sample collection and site description

In this study, activated sludge samples were collected from two bioreactors of different scale: one is a full-scale wastewater treatment system and the other is a lab-scale bioreactor which was used to simulate the full-scale bioreactor. The two reactors received identical wastewater and were operated with same process: anaerobic/anoxic/aerobic (A^2^O). Both of them were located in the same WWTP and were operated by the Beijing Drainage Group Co. Ltd.

Activated sludge samples were monthly collected from the aerobic zone of the two bioreactors from May 2010 to April 2011. For archiving, each 1.5 ml sample was dispensed into a 2 ml sterile Eppendorf tube and centrifuged at 14,000×*g* for 10 min. The supernatant was decanted, and the pellets were stored at −20°C prior to analysis. No specific permits were required for the described field studies. We confirm that: i) the locations were not privately-owned or protected in any way; and ii) the field studies did not involve endangered or protected species.

### DNA extraction

The pellets of the activated sludge samples were washed three times by centrifugation using sterile high-purify water for 5 min at 15,000×*g*. DNA was extracted using a FastDNA SPIN Kit for Soil (MP Biotechnology, USA) according to the manufacturer's protocol.

### PCR amplification and purification

The extracted DNA samples were amplified with a set of primers targeting the hypervariable V4 region of the 16S rRNA gene. The forward primer is 5′-AYTGGGYDTAAAGNG-3′ and the reverse primers are an equal portion mixture of four primers, i.e. 5′-TACCRGGGTHTCTAATCC-3′, 5′-TACCAGAGTATCTAATTC-3′, 5′-CTACDSRGGTMTCTAATC-3′, and 5′-TACNVGGGTATCTAATCC-3′ [20], [21]. Barcodes that allow sample multiplexing during pyrosequencing were incorporated between the 454 adapter and the forward primer.

### Pyrosequencing and sequence analysis

The composition of the PCR products of V4 region of 16S rRNA genes was determined by pyrosequencing using the Roche 454 FLX Titanium sequencer (Roche, Nutley, NJ, USA). Samples in this study were individually barcoded to enable multiplex sequencing. The raw reads have been deposited into the NCBI Sequence Read Archive (Accession Number: SRR952788 and SRR954283). After pyrosequencing, Python scripts were written to remove sequences containing more than one ambiguous base (‘N’) and the sequences shorter than 150 bps. The RDP Classifier (Version 10.31) was used to assign all effective sequences to taxonomic ranks with a set confidence threshold of 50%.

### Data analysis

Temporal variation of bacterial taxa was assessed graphically by the ordination method of nonmetric multidimensional scaling (NMDS) using a statistical software PC-ORD version 6 (MJM Software Design, Gleneden Beach, OR, USA). In the present analysis, a matrix of the OTUs for each sample was used as input data. NMDS ordination was generated based on the Sorenson similarity matrix which was constructed for all pairs of samples.

Moving-window analysis was used to characterize the change rate of bacterial community in this study [22]. Firstly, a similarity matrix for each activated sludge sample was calculated based on Pearson product-moment correlation coefficient using SPSS version 17.0 software (IBM Corporation, Chicago, IL, USA). Then, each similarity percentage value was subtracted from the 100% similarity value to get the change values. Finally, moving-window analysis was performed by plotting the change values between month x and month x−1 [23]. The average change value Δt (one month) was calculated as the average and standard deviation for the respective change values [4].

Canonical Correspondence Analysis (CCA) was used to reveal relationships between bacterial community dynamics and operational and environmental parameters. Statistically important explanatory variables were identified by the forward selection method using a Monte Carlo permutation test (499 permutations under the full model). Operational variables that failed to contribute significant improvement (P<0.05) to a model's explanatory power were excluded from final CCA analyses. The contributions of wastewater characteristics (W), operational parameters (O) and scale of bioreactor (S) to the variances of bacterial communities were assessed with variance partitioning analysis (VPA) using CCA. All wastewater characteristics, operational parameters, and bioreactor scale data were log_2_ (x+1) transformed for standardization. CCA and VPA were performed using the VEGAN package in R (v.2.15.1; http://www.r-project.org/)

## Results

### Bioreactor performance and operational conditions

For the one year period (May 2010 to April 2011), 13 operational and environmental parameters were monitored from the two activated sludge bioreactors. Variations in these parameters are summarized in Table S1 and S2. Performance of the full-scale bioreactor was relatively stable across the sampling period. Although biological oxygen demand (BOD) in the influents varying from 150 to 288 mg/L, the BOD removal efficiency was always excellent (>93%) over the duration of study. The average BOD concentration in the effluent was below 9 mg/L. Total nitrogen (TN) in the influent ranged from 35.9 to 69.3 mg/L, while the ammonium concentration was between 32.8 and 54.2 mg/L. The average removal efficiencies of TN and ammonia were 62% and 97%, giving final concentrations in effluent of less than 20.9 mg/L and 1.5 mg/L, respectively. The temperature of the bioreactor showed a seasonal pattern from 16.3°C (winter) to 25.1°C (summer). The bioreactor was maintained with relatively stable pH (7.1±0.3), hydraulic residence time (HRT) (8.2±1.3 h), DO (3.5±0.5 mg/L) and mixed liquor suspended solids (MLSS) (3,775±640 mg/L), while solid retention time (SRT) (8.7±2.1 days) exhibited a larger variation comparatively.

The lab-scale bioreactor was also relatively functional stable during the study period. The influent characteristics were the same as the full-scale bioreactor. The average BOD, TN and ammonia removal capacities were 94%, 61%, and 97%, giving final average concentrations in effluent of less than 7 mg/L, 22 mg/L and 2 mg/L, respectively. Other environmental and operational parameters (pH, DO, MLSS, HRT and SRT) were kept relatively stable (Table S2).

### Bacterial community composition

By using 454 pyrosequencing, 13422–31151 effective sequence tags were yielded for the 24 samples. The library size of each sample was normalized to 13422 sequences by randomly removing the extra sequences, which was the smallest number of sequencing reads among the 24 samples, to conduct the downstream analyses for different samples at the same sequencing depth.

RDP Classifier was used to assign these sequence tags into different OTU with 3% of nucleotide cutoff. A total of 10223 OTUs were recovered from the full-scale bioreactor and 14791 OTUs were from the lab-scale bioreactor. Individual samples contained much smaller number of OTUs from 2513 to 3878 in the full-scale bioreactor and 2137 to 3275 within the lab-scale bioreactor (Table 1).

**Table 1:** Number of taxa classified by different taxonomic levels from the full-and lab-scale bioreactor.

Taxonomic level	bioreactor	The number of taxa at each level												Range	Average	Standard deviation
		1	2	3	4	5	6	7	8	9	10	11	12			
Phylum	Full^a^	12	13	13	16	16	14	16	15	14	14	15	14	12–16	14.3	1.2
	Lab^b^	12	13	14	15	16	14	16	15	16	14	15	14	12–16	14.5	1.2
Class	Full	23	23	26	29	31	28	27	28	23	26	26	27	23–29	26.4	2.4
	Lab	21	24	27	23	32	29	29	22	23	24	21	23	21–32	24.8	3.4
Order	Full	51	53	52	64	65	61	57	57	56	50	51	51	50–65	55.7	5.1
	Lab	49	60	62	58	66	64	64	53	57	53	47	51	49–66	57.0	6.1
Family	Full	65	78	67	84	92	76	62	73	64	63	68	65	63–92	71.4	9.0
	Lab	63	71	73	79	87	70	69	62	78	65	59	64	59–87	70.0	7.9
Genus	Full	219	274	231	278	281	256	249	237	215	201	229	205	201–281	239.6	26.8
	Lab	208	242	251	267	278	245	231	205	261	237	197	223	197–278	237.1	24.4
OTUs	Full	3611	3043	2787	3878	3592	3317	2706	2847	3452	3027	2779	2513	2513–3878	3055.8	408.0
	Lab	2454	2983	3150	3275	3742	2773	2870	2697	3130	2993	2137	2452	2137–3275	2874.8	426.2

a Full-scale bioreactor.

b Lab-scale bioreactor.

RDP Classifier was also used to assign these sequence tags into different phylogenetic bacterial taxa. The threshold for the bootstrap cutoff was set to 50% to ensure the classification reliability [12]. Table 1 summarizes the numbers of taxa at different levels of classification within the two bioreactors. The bacterial community composition of the two bioreactors showed typical activated sludge communities [21], [24], [25]. At the phylum level, *Proteobacteria* was the predominant phylum in the full-scale bioreactor, constituting between 19 and 51% of all detected OTUs. *Bacteroidetes*, *Acidobacteria*, and *Chloroflexi* were the subdominant groups, each containing 13–52%, 1–22% and 1–14% of the detections respectively. These four phyla represented approximately 73–95% of bacteria detected within the full-scale bioreactor. Within *Proteobacteria* the *β*-subdivision was the predominant group (26–54%), followed by *α-Proteobacteria* (6–43%), *γ-Proteobacteria* (7–37%) and *δ-Proteobacteria* (1–19%). Within the *β-Proteobacteria*, six taxa were identified. *Rhodocyclales* is the dominant group within a range of 25–68% of all 12 samples, followed by *Burkholderiales* and *Burkholderiales*, representing 14–52% and 8–32% of each population respectively. The other three detected groups (*Burkholderiales*, *Rhodocyclales*, and *Burkholderiales*) had fewer detections in all samples and constituted less than 10% of *β-Proteobacteria*.

Similar to the full-scale bioreactor, in the lab-scale bioreactor *Proteobacteria* was also the predominant phylum (22–49%), following *Bacteroidetes* (17–43%), *Acidobacteria* (2–23%), *Chloroflexi* (1–19%). Within *Proteobacteria β*-subdivision was the predominant group (17–49%), followed by *α-Proteobacteria* (7–46%), *γ-Proteobacteria* (5–39%) and *δ-Proteobacteria* (3–25%).

### Tracking bacterial community dynamics

**Figure 1:**
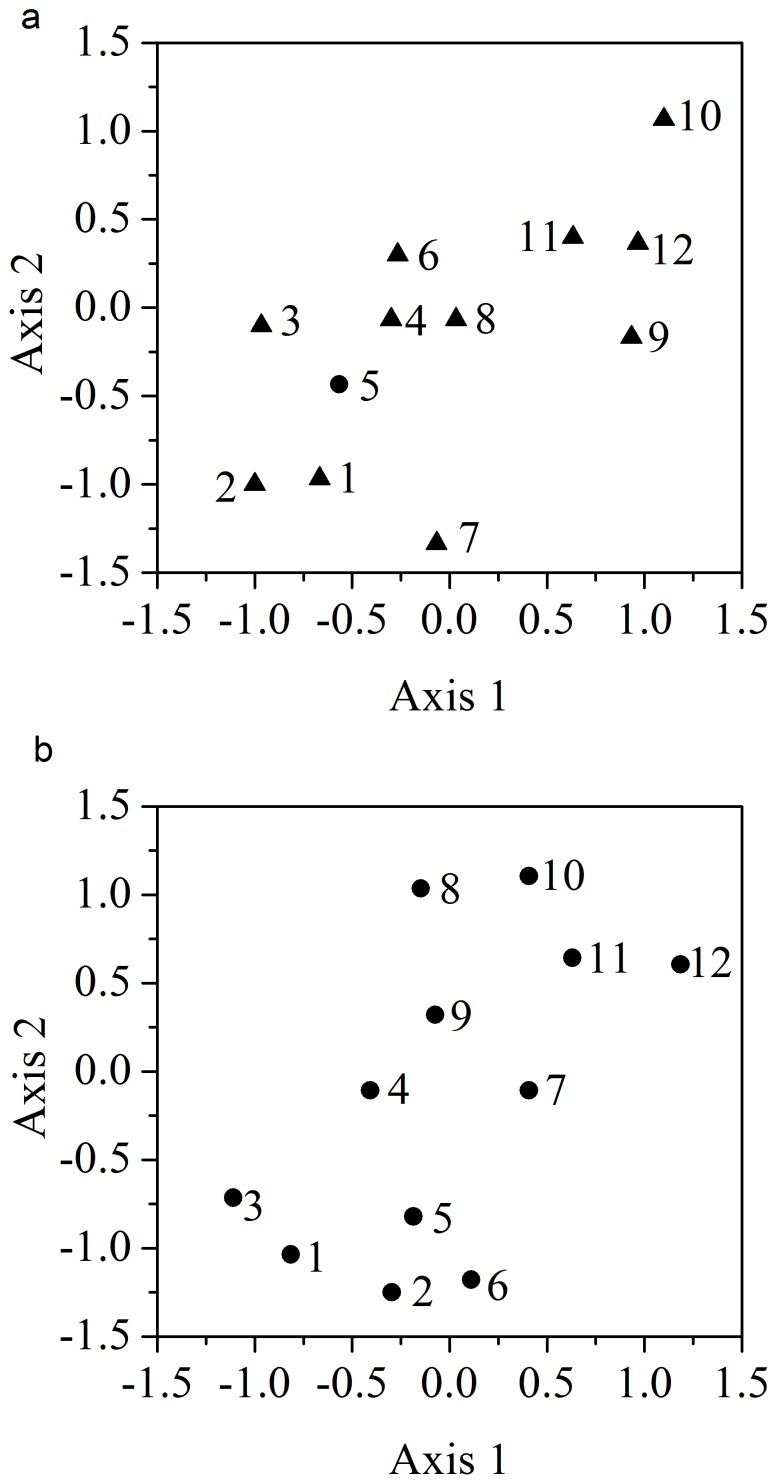
Ordination of nonmetric multidimentional scaling based on OTUs (3% cutoff) identified from a full- (a) and lab-scale (b) bioreactors. Sample numbers: 1- May 2010, 2- June 2010, 3-July 2010, 4- August 2010, 5-September 2010, 6- October 2010, 7-November 2010, 8-December 2010, 9-January 2011, 10- February 2011, 11-March 2011, 12-April 2011.

The temporal dynamics of the bacterial compositions within the two bioreactors was evaluated using an indirect gradient ordination technique nonmetric multidimensional scaling (NMDS) ordination (Fig. 1) which was constructed based on the observed OTUs at a 3% cutoff. At a two-dimensional solution, the stress values of the ordination for the full- and lab-scale reactors were 16.7 and 14.8, respectively, demonstrating that the reduced NMDS ordinates preserve patterns in activated sludge bacterial community dynamics. In general, samples collected in similar time periods were clustered closely together in the ordination. This ordination pattern suggests a gradual succession within the overall bacterial community over time.

**Figure 2:**
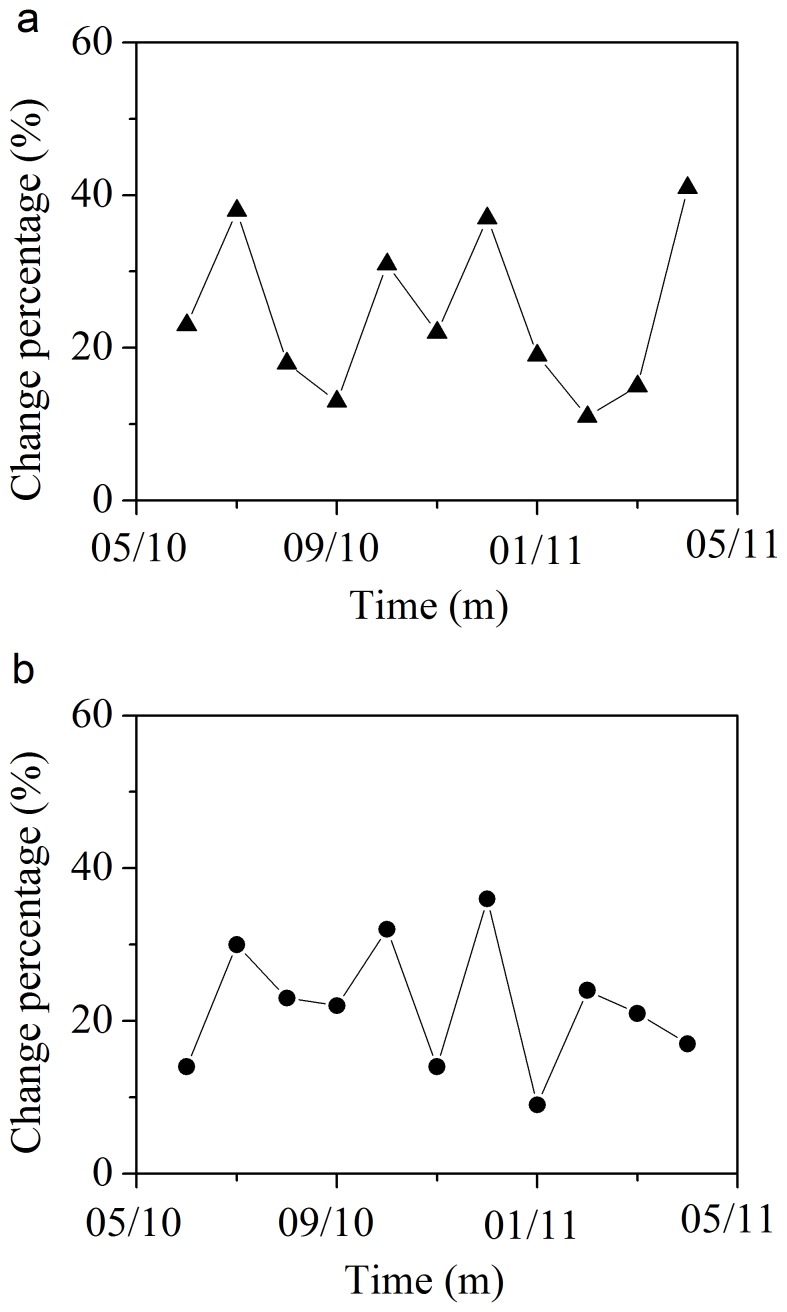
Moving-window analysis based on pyrosequencing data for a full-scale (a) and lab-scale (b) bioreactors. Each data point in the graph is a comparison between two consecutive dates, as it represents the correlation between the samples of month x and month x−1.

Moving window analysis was used to evaluate the change rate of bacterial community [26]. The correlation coefficients of two consecutive dates (one month) from the full-scale bioreactor were between 64% and 91%. Thus, changes were from 9% to 36% (Fig. 2), and the Δ*_t_*_ (one month)_ was 21.4%±9.5%. While the change rate for the lab-scale bioreactor were 13%–41%, with the Δ*t*_(one month)_ 25.1%±11.7%, which is higher than the full-scale bioreactor.

### Correlations between bacterial community dynamics and operational and environmental variables

**Figure 3:**
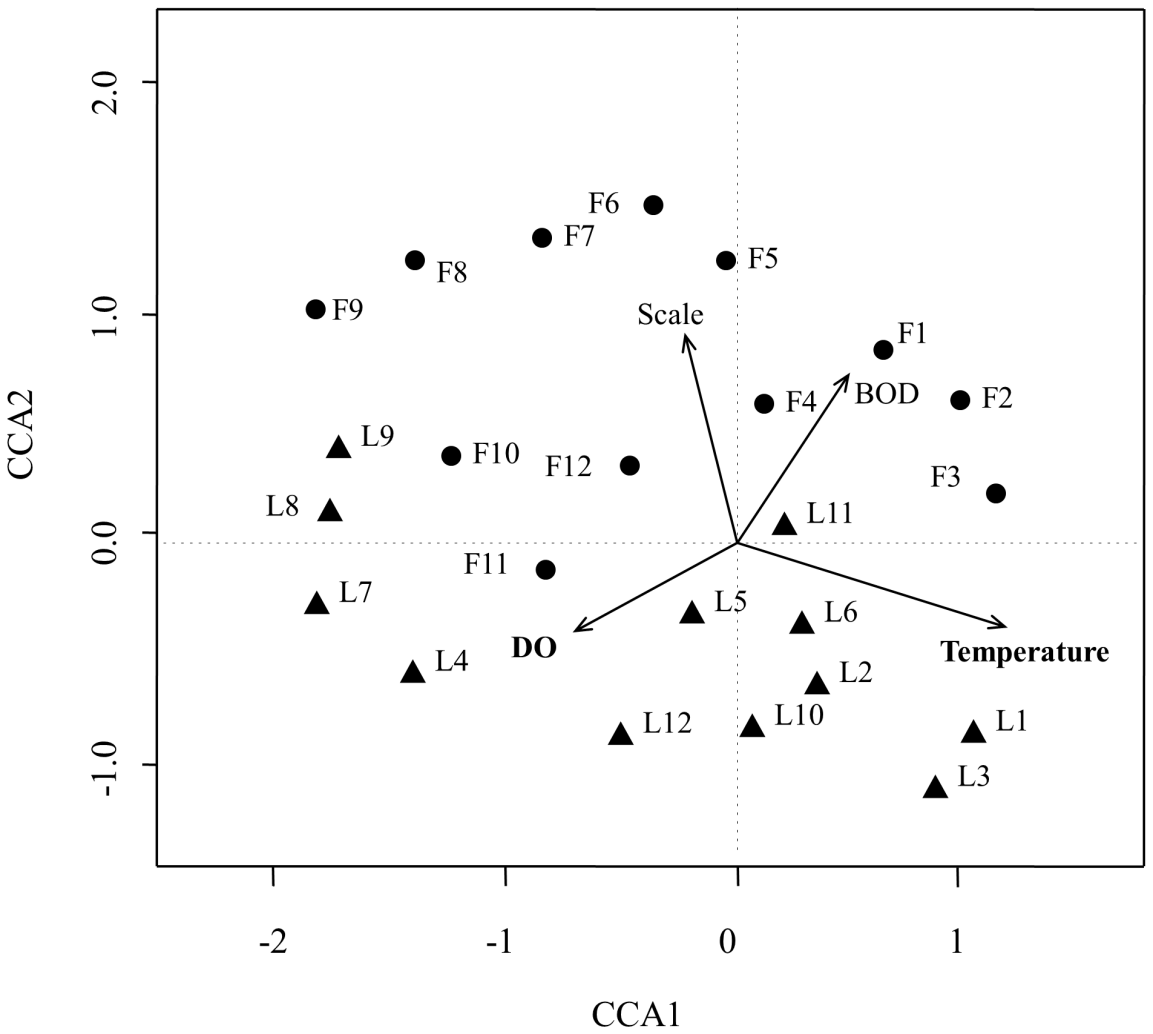
Canonical correspondence analysis (CCA) of pyrosequencing data and measurable variables in a full- and lab-scale bioreactors. Arrows indicate the direction and magnitude of measurable variables associated with bacterial community structures. Circles and triangles represent different bacterial community structures from the full- and lab-scale bioreactor, respectively. Samples are named with “F” (Full-scale bioreactor) or “L” (Lab-scale bioreactor) and numbers. Sample numbers: 1- May 2010, 2- June 2010, 3-July 2010, 4- August 2010, 5-September 2010, 6- October 2010, 7-November 2010, 8-December 2010, 9-January 2011, 10- February 2011, 11-March 2011, 12-April 2011.

CCA was performed to discern the possible relationship between bacterial community structure and operational and environmental variables (Fig. 3). Based on variance inflation factors (VIF) with 999 Monte Carlo permutations, four significant environmental variables: temperature, influent BOD, bioreactor scale and DO were selected in the CCA biplot. The length of an environmental parameter arrow in the ordination plot indicates the strength of the relationship of that parameter to community composition. As such, temperature, influent BOD, DO and Bioreactor scale appears to be the most important environmental parameters.

**Figure 4:**
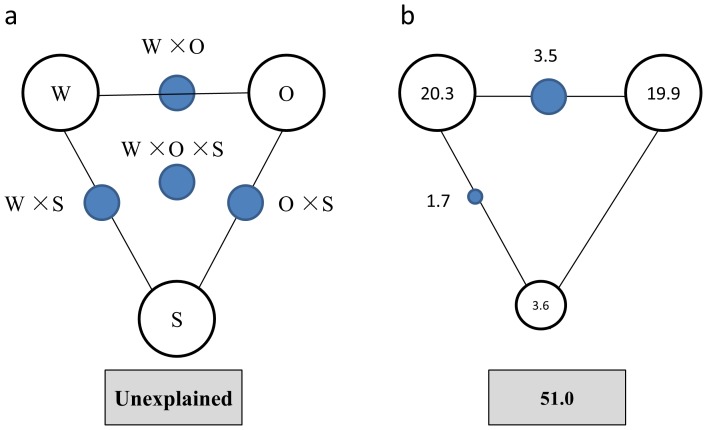
Variation partitioning analysis of microbial community explained by wastewater characteristics (W), operational parameters (O), and Scale of bioreactor (S). (a) General outline; (b) bacterial communities. Each diagram represents the biological variation partitioned into the relative effects of each factor or a combination of factors, in which geometric areas were proportional to the respective percentages of explained variation. The edges of the triangle represent the variation explained by each factor alone. The sides of the triangles represent interactions of any two factors, and the middle of the triangles represent interactions of all three factors.

VPA was further performed to assess the contributions of wastewater characteristics (BOD, TN, TP, pH), operational parameters (temperature, SRT, HRT, MLSS), and bioreactor scale to the bacterial community variance. Fig. 4 indicated that 49% of the variance could be explained by these three components. Wastewater characteristics, operational parameters and bioreactor scale could independently explain 20.3%, 19.9%, and 3.6% of the variation of bacterial communities, respectively. Interactions among the three major components seemed to have less influence than did individual components, and were only observed between wastewater characteristics and operational parameters (3.5%) and between wastewater characteristics and bioreactor scale (1.7%).

### Taxa-time relationship (TTR)

**Figure 5:**
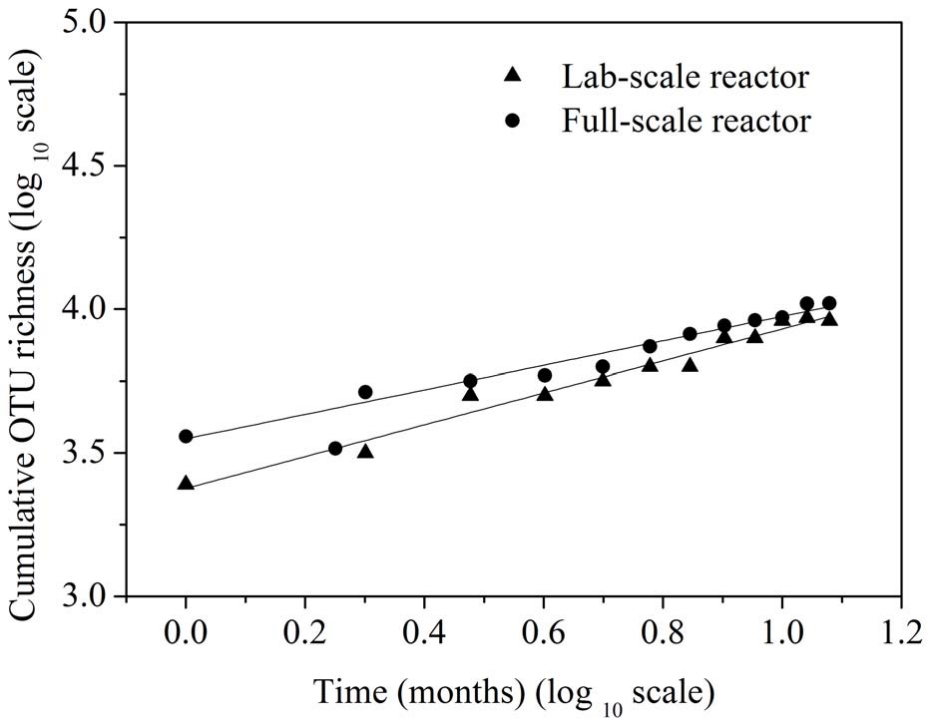
Taxa-time relationships for a full-scale bioreactor (circles) and lab-scale bioreactor (triangles). The lines are fitted to a power law equation *S* = *cT^w^*, where *S* is the number of observed taxa, *c* is the constant, *T* is the time, and *w* is the taxa-time relationship exponent.

The bacterial TTRs for the two bioreactors were visualized using the power law equation *S* = *cT^w^*, as described above, and plotted in log-log space in Fig. 5. The TTR relationship exponent (*w*) is the slope of the linear regression line fitted to the log–log plots, which can be considered a measure of temporal turnover of microbial taxa. The exponents (i.e., temporal turnover rate) for the full-scale bioreactor was 0.43 (*R^2^* = 0.970), which is lower that of the lab-scale bioreactor (*w* = 0.55, *R^2^* = 0.971).

The TTRs for phyla level was further analyzed. The results showed that the power law exponents of 13 phyla ranged from 0.32 to 0.57 in the two bioreactors (Table 2), suggesting that bacterial phyla have different temporal turnover rates. Interestingly, the exponents of dominant phyla were general lower than that of the rare phyla (Table 2). For example, the average exponents of five most dominant phyla (*Proteobacteria*, *Bacteroidetes*, *Acidobacteria*, *Chloroflexi* and *Verrucomicrobia*) within the full- and lab-scale bioreactor were 0.35 and 0.36, while that of the five rarest phyla (*Synergistetes*, *Fusobacteria*, *TM7*, *Euryarchaeota* and *OP10*) were 0.50 and 0.55.

**Table 2:** The power law exponent of taxa-time relationship for each phylum within two activated sludge bioreactors.

Phyla	Full-scale bioreactor			Lab-scale bioreactor		
	*Relative abundance (%)* a	*w*	*r^2^*	*Relative abundance(%)*	*w*	*r^2^*
*Proteobacteria*	25.8	0.33	0.951	43.1	0.34	0.954
*Bacteroidetes*	41.0	0.32	0.965	27.1	0.35	0.955
*Acidobacteria*	11.7	0.37	0.952	4.6	0.36	0.944
*Chloroflexi*	1.9	0.38	0.949	9.0	0.37	0.983
*Verrucomicrobia*	5.6	0.37	0.968	8.5	0.38	0.957
*Planctomycetes*	1.8	0.42	0.967	1.1	0.48	0.962
*Firmicutes*	2.5	0.42	0.975	3.7	0.46	0.967
*Actinobacteria*	0.3	0.41	0.971	0.6	0.47	0.971
*Chlamydiae*	0.6	0.46	0.954	0.3	0.52	0.966
*Spirochaetes*	0.6	0.46	0.969	0.4	0.54	0.958
*Synergistetes*	0.1	0.49	0.972	0.1	0.54	0.973
*Fusobacteria*	0.1	0.47	0.955	0.1	0.55	0.982
*TM7*	0.1	0.52	0.967	0.1	0.57	0.981
*Euryarchaeota*	0.1	0.53	0.981	0	0.55	0.979
*OP10*	0.2	0.51	0.977	0.1	0.56	0.978

a It is an average of the relative abundance of each phylum within 12 samples.

## Discussion

Understanding the factors that shape microbial community structure in WWTPs could potentially enhance treatment performance and control. In this study, CCA ordination analysis indicated that temperature was an important variable influencing microbial community structures. This agrees with the findings of Wells et al. [5], who suggested, based on the survey of bacteria community variance via terminal restriction fragment length polymorphism (T-RFLP) in a full-scale WWTP, that water temperature is one of the most influential variables on bacterial community variance. Similar results have also been obtained in an expanded granular sludge bed (EGSB) bioreactor by Siggins *et al.* [27]. As with temperature, influent BOD was also significantly linked to bacterial community structures. BOD provides carbon and energy sources to heterotrophic bacteria and influences the growth rate of the bacteria [12]. Previous studies have reported that BOD (or organic loading) was an important factor to mediate the bacterial community structures [28]. In addition to temperature and influent BOD, DO was also strongly and significantly linked to bacterial community variance in CCA analyses. DO is well recognized as a critical process parameter in biological wastewater treatment processes, due to its impact on bacterial activity and the high operational costs of aeration, but little is known about the specific selection of distinct bacterial lineages by DO concentration [29]. The results of this study showed that DO had a significant effect in shaping the bacterial community structure in wastewater treatment systems. In two lab-scale bioreactors with high and low DO concentrations, Park *et al.* [30] have also demonstrated that DO concentration was an important structuring factor based on the T-RFLP analysis of bacterial community structures. Bioreactor scale is also one of the important factors mediating the bacterial community. To data, there are rare studies have examined the effect of bioreactor scale on the microbial community structure and diversity. In seven membrane bioreactors (MBR) of increasing size, van der Gast *et al.* [31] observed a significant linear relationship between bacterial taxa richness and reactor size, and they also found a gradient of greater evenness in community structure as MBR volume increased. Additional research is warranted to establish a firm understanding of how bioreactor sizes affect microbial communities in activated sludge systems.

The VPA results showed that 49% of the community variances were explained by these three components. Thus 51% of the community variance could not be explained by these three components. It is reasonable to expect that some unmonitored wastewater and operational variables may play an influential role in mediating bacterial community structures in WWTPs. In addition to the deterministic factors (wastewater and operational variables), the neutral factors (random immigration and births/deaths) may also affect the structure of the bacterial community. Ofiteru *et al.* [9] demonstrated that, in a full-scale WWTP, the variation of bacterial community was consistent with neutral community assembly, where chance and random immigration played an important and predictable role in shaping the communities. In four activated sludge bioreactors, Ayarza and Erijman [32] illustrated that both neutral and deterministic effects operated simultaneously in the assembly of bacterial floc. Similar results were also observed in other studies [33], [34] [35]. The relative influence of deterministic environmental and stochastic factors in structuring microbial communities within bioreactors warrants future investigation.

We demonstrated that accumulation of observed bacterial taxa in activated sludge over time followed a power-law relationship, with a power-law exponent of *w* = 0.43 and 0.55 for a full- and lab-scale bioreactors, respectively. The values fell within the typical values determined previously. Shade *et al.* [16] conducted a meta-analysis of temporal dynamics in microbial communities, including 76 sites representing air, aquatic, soil, brewery wastewater treatment, human- and plant-associated microbial biomes. They found that there was a very consistent TTR across microbial communities, and the power law exponents ranged between 0.24 and 0.61. Our observed scaling exponent is also well in line with the few previous studies that have examined TTRs of bacterial taxa in activated sludge systems. van der Gast et al. [11] employed lab-scale reactors to examine the impact of different percentages of municipal and industrial wastewater on TTR exponent, and found that the power law exponent decreased pronounced from 0.512 to 0.162 as selective pressure (industrial wastewater concentration) increased. Wells *et al.* [10] detailed a power law exponent of *w* = 0.209 for bacterial communities in a full-scale WWTP, based on T-RFLP of bacterial 16S rRNA gene.

In our study, the power law exponents of dominant phyla were general lower than that of the rare phyla. Similar results were also obtained by Kim *et al.* [12], who reported that in a full-scale activated sludge bioreactor, the exponents for the general and rare bacterial taxa were 0.23 and 0.55, respectively.

The power law exponent of the full-scale bioreactor is lower than the value for the lab-scale bioreactor. It is tempting to speculate that this discrepancy is associated with the more than five order of magnitude variation in volume between these two reactors. Indeed, White *et al.* [18] has demonstrated that the exponent of TTR in macroecology was negatively correlated the spatial scale of observation, and van der *et al.* [11] suggested that this may be the case in microecolgy as well. Moreover, recent work has demonstrated the interdependence of spatial and temporal accumulation of species in the species-time-area relationship (STAR) in various systems [36]. Additional research is warranted to study the relationship between spatial and temporal turnover in microbial systems.

In conclusion, our results showed that accumulation of observed taxa in activated sludge over time followed a power-law relationship. The power-law exponent *w* for the full-scale bioreactor was 0.43, which is lower that of the lab-scale bioreactor (*w* = 0.55). The power law exponents of dominant phyla were general lower than that of the rare phyla within the two bioreactors. Overall, our results suggest that bacterial communities of activated sludge exhibited TTRs similar to those observed previously for plant and animal communities. These results highlight that a continued integration of microbial ecology into the broader field of ecology.

## Supporting Information

Table S1 **Operational conditions and bioreactor performance of the full-scale bioreactor.**
(DOCX)

Table S2 **Operational conditions and bioreactor performance of the lab-scale bioreactor.**
(DOCX)
