# Supplementary material for: Bacterial Community Dynamics and Taxa-Time Relationships within Two Activated Sludge Bioreactors
Source: PLoS One. 2014 Mar 4;9(3):e90175. doi: 10.1371/journal.pone.0090175 (PMC3942418; doi:10.1371/journal.pone.0090175)
Supplement: Table S1 — Operational conditions and bioreactor performance of the full-scale bioreactor. (DOCX) [file pone.0090175.s001.docx]

Table S1 Operational conditions and bioreactor performance of the full-scale bioreactor

| Characteristics | Number of  measurement | Range | Average | Standard deviation |
| --- | --- | --- | --- | --- |
| Wastewater inflow rate (m^3^/d) | 365 | 17693-28932 | 21507 | 2297 |
| Mixed liquor temperature (℃) | 365 | 16.3-25.1 | 21.3 | 1.9 |
| Dissolved oxygen (mg/L) | 365 | 2.3-4.9 | 3.5 | 0.5 |
| Mixed liquor suspended solids (mg/L) | 52 | 2787-5423 | 3775 | 640 |
| pH | 365 | 6.4-7.6 | 7.1 | 0.3 |
| Hydraulic retention time (hr) | 365 | 6.7-11.1 | 8.2 | 1.3 |
| Solids retention time (day) | 52 | 4.8-16.9 | 8.7 | 2.1 |
| Influent BOD5 (mg/L) | 104 | 150-288 | 197.3 | 36.3 |
| Effluent BOD5 (mg/L) | 104 | 2.5-12.8 | 7.1 | 2.1 |
| Influent TN (mg/L) | 104 | 35.9-69.3 | 53.8 | 7.7 |
| Effluent TN (mg/L) | 104 | 19-30.7 | 20.9 | 3.1 |
| Influent ammonia (mg/L) | 104 | 32.8-54.2 | 43.6 | 4.9 |
| Effluent ammonia (mg/L) | 104 | 0.2-2.6 | 1.5 | 0.6 |
