# Supplementary material for: Bacterial Community Dynamics and Taxa-Time Relationships within Two Activated Sludge Bioreactors
Source: PLoS One. 2014 Mar 4;9(3):e90175. doi: 10.1371/journal.pone.0090175 (PMC3942418; doi:10.1371/journal.pone.0090175)
Supplement: Table S2 — Operational conditions and bioreactor performance of the lab-scale bioreactor. (DOCX) [file pone.0090175.s002.docx]

Table S2 Operational conditions and bioreactor performance of the lab-scale bioreactor

| Characteristics | Number of  measurement | Range | Average | Standard deviation |
| --- | --- | --- | --- | --- |
| Wastewater inflow rate (m^3^/d) | 365 | 0.86-1.25 | 0.96 | 0.2 |
| Mixed liquor temperature (℃) | 365 | 16.7-24.9 | 21.1 | 1.8 |
| Dissolved oxygen (mg/L) | 365 | 2.5-4.1 | 3.3 | 0.4 |
| Mixed liquor suspended solids (mg/L) | 52 | 2945-4973 | 3548 | 517 |
| pH | 365 | 6.7-7.6 | 7.2 | 0.3 |
| Hydraulic retention time (hr) | 365 | 6.9-10.1 | 7.8 | 0.8 |
| Solids retention time (day) | 52 | 5.3-13.1 | 8.5 | 1.9 |
| Influent BOD5 (mg/L) | 104 | 150-288 | 197.3 | 36.3 |
| Effluent BOD5 (mg/L) | 104 | 2.2-11.3 | 6.9 | 2.0 |
| Influent TN (mg/L) | 104 | 35.9-69.3 | 53.8 | 7.7 |
| Effluent TN (mg/L) | 104 | 19-31.6 | 21.8 | 3.3 |
| Influent ammonia (mg/L) | 104 | 32.8-54.2 | 43.6 | 4.9 |
| Effluent ammonia (mg/L) | 104 | 0.3-2.9 | 1.6 | 0.6 |
